# Reproducibility and Reliability Of QTc and QTcd Measurements and Their Relationships with Left Ventricular Hypertrophy in Hemodialysis Patients

**DOI:** 10.5935/abc.20170112

**Published:** 2017-09

**Authors:** Maria Angélica Gonçalves Alonso, Valentine de Almeida Costa de Castro Lima, Maria Angela Magalhães de Queiroz Carreira, Jocemir Ronaldo Lugon

**Affiliations:** 1 Universidade Federal Fluminense (UFF), Niterói, RJ - Brazil; 2 Hospital Universitário Antônio Pedro - Universidade Federal Fluminense (UFF), Niterói, RJ - Brazil

**Keywords:** Electrocardiography, Hypertrophy, Left Ventricular, Coronary Artery Disease, Cardiomyopathy, Hypertrophic, Renal Dialysis

## Abstract

**Background:**

Left ventricular hypertrophy (LVH) is very common in hemodialysis patients and an independent risk factor for mortality in this population. The myocardial remodeling underlying the LVH can affect ventricular repolarization causing abnormalities in QT interval.

**Objective:**

to evaluate the reproducibility and reliability of measurements of corrected QT interval (QTc) and its dispersion (QTcd) and correlate these parameters with LVH in hemodialysis patients.

**Methods:**

Case-control study involving hemodialysis patients and a control group. Clinical examination, blood sampling, transthoracic echocardiogram, and electrocardiogram were performed. Intra- and interobserver correlation and concordance tests were performed by Pearson´s correlation, Cohen’s Kappa coefficient and Bland Altman diagram. Linear regression was used to analyze association of QTc or QTcd with HVE.

**Results:**

Forty-one HD patients and 37 controls concluded the study. Hemodialysis patients tended to have higher values of QTc, QTcd and left ventricular mass index (LVMi) than controls but statistical significance was not found. Correlation and concordance tests depicted better results for QTc than for QTcd. In HD patients, a poor but significant correlation was found between QTc and LVMi (R^2^ = 0.12; p = 0.03). No correlation was found between values of QTcd and LVMi (R^2^= 0.00; p=0.940). For the control group, the correspondent values were R^2^= 0.00; p = 0.67 and R^2^= 0.00; p = 0.94, respectively.

**Conclusion:**

We found that QTc interval, in contrast to QTcd, is a reproducible and reliable measure and had a weak but positive correlation with LVMi in HD patients.

## Introduction

Despite the improvement of the quality of dialysis over the years, patients with end-stage renal disease still have a high mortality rate. Heart disease remains the leading cause of death in these patients, with coronary artery disease and left ventricular hypertrophy (LVH) as the most frequent cardiovascular abnormalities. LVH is very common in hemodialysis (HD) patients, and an independent risk factor for mortality in this populaton.^1,2^ Myocardial remodeling is not a homogeneous phenomenon and can affect ventricular repolarization causing non-uniform abnormalities in QT interval (QT).^3^

The QT interval (QT) represents the electrical ventricular systole, and QT dispersion (QTd), defined as the difference between the maximal and minimal QT on a 12-lead electrocardiogram (ECG), reflects the regional heterogeneity of the myocardial repolarization. Several studies have reported an association between increased values of any of these two parameters and all-cause mortality, sudden death, ventricular arrhythmias and coronary artery disease.^4,5^ The measurement of QT is not an easy task and involves a number of pitfalls, as follows: recognizing the onset of the QRS complex and especially the end of the T wave may be difficult; the leads chosen to measure the QT interval varies among studies; there is more than one formula to adjust the QT interval for the cardiac rate; and finally, cut-off values for both QT and QTd are not well defined and the role of gender adjustment in this regard is disputable.^6,7^

While ECG is available in almost every dialysis center, the echocardiogram (ECO), considered the gold standard for the diagnosis of LVH, is not. In view of that we thought it would be of interest to investigate the reproducibility and reliability of corrected QT (QTc) and its dispersion (QTcd) measurements and their relationships with LVH in HD patients.

## Methods

### Study population

This study used the database generated by a previous study.^8^ The protocol was approved by the ethics committee of the university medical school under the number 0125.0258.000-10/2010 and a written informed consent was obtained from every patient. We conducted a case-control study with HD patients recruited from a single dialysis center and a control group matched by gender and age without overt kidney disease. HD patients should be on treatment for at least 3 months, in a schedule of 4-hour duration sessions, 3 times a week. The control group consisted of individuals referred for exercise testing at the university hospital. Participants should be aged between 18 and 70 years. Exclusion criteria were as follow: arrhythmias that prevent proper assessment of heart rate, presence of symptomatic heart disease, and, in the control group, an estimated glomerular filtration rate by the CKD-EPI equation^9^ lower than 60 ml/min/1.73 m^2^. Regular medications were not discontinued for the study. Cardiac evaluation was performed in the interval between dialysis sessions, in the middle of the week, and consisted of clinical examination, transthoracic ECO, and ECG. Blood samples were collected before the HD procedure for determination of ultrasensitive C-reactive protein and hemoglobin. The urea reduction ratio (URR) was calculated as the average of the last three determinations prior to enrollment. In the control group, blood sample collection (for determination of C-reactive protein, creatinine and hemoglobin levels) and cardiac evaluation were performed 30 min before the exercise test. C-reactive protein was analyzed by an immunoturbidimetric assay (Dimension RxLMax, Siemens, Berlin, Germany).

### Echocardiography

A two-dimensional transthoracic ECO was performed with GE VIVID 7 System (General Electric Company, USA) by an experienced echocardiographist without prior knowledge of the results of other tests. Determination of internal chamber size, global and segmental ventricular systolic function, diastolic function and structural changes were performed. Patients and controls were considered to have LVH if left ventricular mass index (LVMi) were higher than 88 g/m^2^ in women and 102 g/m^2^ in men.^10^

### Electrocardiogram and QT measurement

A 3-channel recorder was used for the electrocardiographic traces (Ergo 13, Heart Ware Co., Minas Gerais, Brazil). The twelve electrocardiographic leads were recorded on paper at a speed of 25 mm/s with patients at rest. Two observers (unaware of each other’s results) manually measured the QT and its dispersion on the same electrocardiographic traces at two different times with an interval of one week between measurements. QTs were measured using the method of the tangent,^11^ in which the end of the T wave is defined at the intersection point of the tangent line, drawn at the point of greatest slope of the last portion of the T wave, with the baseline. In the presence of the U wave, the tangent was drawn crossing the meeting point between the U and T waves. The chosen leads were DII or V5 (which had the highest value of QT) and the cutoff value for an enlarged QTc was ≥ 450 ms for men and ≥ 460 ms for women.^12^ Leads in which a tangent could not be drawn because of unclear definition of T wave morphology were excluded from analysis. The correction of the QT for heart rate was performed by the method of Hodges^12^ with the formula: QTc = QT + 1.75 (RR interval - 60). QT dispersion was obtained as usual, i.e. as the difference between the highest and the lowest QT value on a 12 lead ECG. Values of QTcd > 60 ms were considered abnormal.^13,14^

### Statistical analysis

Results were expressed as mean and standard deviation for normally distributed data and median and range otherwise. Categorical variables were expressed as frequencies and compared using the Fisher Test. Comparisons between two continuous variables were accomplished by the non-paired T test (for normal distribution) or its nonparametric equivalent (Mann-Whitney test). For evaluation of the reproducibility and reliability of QTc and QTcd measures, intra and inter observer agreement, and concordance tests were performed employing Pearson´s correlation, Cohen's Kappa coefficient and Bland Altman diagram, respectively. Linear regression was used to analyze association of QTc and QTcd with LVH. p < 0.05 was considered significant. Analyses were performed using SPSS for Windows version 18.0 (SPSS Inc., Chicago, IL, USA) and MedCalcversion 16.4.3 (Medcalcsoftware bvba, Belgium).

## Results

From a total of 125 patients from a single dialysis center, after application of exclusion criteria, 51 agreed to participate and signed the consent form. Ten patients did not show up for the exams resulting in 41 HD patients that concluded the study. From 41 control patients initially selected, 4 were excluded: 2 due to incomplete data and 2 had estimated glomerular filtration rate below 60 mL/min/1.73 m^2^. Data for LVMi were available in 38 HD patients and 30 controls. The general features of participants are in Table 1. The most common etiologies of the renal disease were: hypertensive nephrosclerosis (56%), chronic glomerulonephritis (17%), polycystic kidney disease (10%), and diabetic nephropathy (7%).

**Table 1:** General features of 41 patients and 37 controls and echocardiogram data available in 38 patients and 30 controls

	Hemodialysis patients	Controls	p value
Age, years	50 ± 14^a^	50 ± 12	0.975
Male gender (%)	21 (51.2)	18 (48.6)	0.145
Non-white (%)	27 (65.9)	18 (48.6)	0.402
Body mass index, kg/m^2^	25.1 ± 5.1	27.6 ± 4.2	0.016
Dialysis vintage, months	67.2 ± 47.3	n.a	-
Diabetes, (%)	4 (9.8)	4 (10.8)	0.467
Smoking, (%)	3 (9.1)	7 (19)	0.104
Familial CAD, f (%)	15 (36.6)	16 (43.2)	0.669
Familial hypertension, (%)	26 (63.4)	20 (54.1)	0.106
Sedentary, (%)	33 (80.5)	22 (59.5)	0.082
Use of blood pressure drugs (%)	33 (80.5)	19 (51.4)	0.860
Beta-blocker	14 (34.1)	6 (16.2)	0.411
Diuretic	2 (4.9)	8 (21.6)	0.599
Calcium channel blocker	5 (12.2)	2 (5.4)	0.134
ACE inhibitor/ARB	12 (29.3)	15 (40.5)	0.433
Clonidine	8 (19.5)	0	< 0,001
Alfa-blocker	6 (14.6)	0	< 0.001
C-reactive protein, mg/dL	1.02 ± 1.20	0.5 ± 0.52	0.016
URR, %	68.7 ± 7.8	n.a.	-
Hemoglobin, g/dL	11.5±1.4	13.8 ± 1.2	< 0.001
Left ventricular mass index, g/m^2^	128 ± 52	107 ± 30	0.054
Left ventricular hypertrophy, % ^b^	71	46	0.118
QTc, ms	418 ± 29	407 ± 27	0.085
QTcd, ms	57 ± 22	50 ± 20	0.189
Enlarged QTc^c^, %	15	5.4	0.268
QTcd > 60 ms, %	34	21	0.314

a Mean ± S.D.;

b > 110 g/m^2^ for male and >88 g/m^2^ for female;

c ≥ 450 msec for male and ≥ 460 msec for female; ACE: angiotensin-converting–enzyme; ARB - AT1: receptor blocker; CAD: coronary artery disease; URR: urea reduction ratio; QTc: corrected QT interval; QTcd: Dispersion of QTc. Differences between continuous variables were tested by non-paired T test; For categorical variables, the Fisher Test was employed.

Systolic function of the left ventricle, as analyzed by the ejection fraction, was similar between groups (66.1 ± 10.1% *vs.* 68.6 ± 5.4% for HD patients and controls, respectively, p = 0.167). The mean LVMi and the prevalence of LVH tended to be higher in HD patients than in controls but statistical significance was not found (128 ± 52 g/m^2^ *vs.* 107 ± 30 g/m^2^, p = 0.054 and 71% *vs.* 46%, p = 0.165, respectively).

Observer 1 excluded for analysis 11 leads at the first measurement and 22 leads at the second one in HD group, and 36 leads and 44 leads at first and second measurement, respectively in the control group. Observer 2 excluded for analysis 13 leads at the first measurement and 18 at the second one in HD group, and 28 and 16 leads at first and second measurements, respectively in the control group.

In HD patients, mean QTc and QTcd measures were 416.6 ± 29.5 ms and 48.3 ± 17.4 ms, respectively by observer 1, and 420.1 ± 30.6 ms and 65.9 ± 30.2 ms for observer 2. In controls, mean values for QTc and QTcd were 408 ± 30.0 ms and 47 ± 17.3 ms for observer 1 and 406.2 ± 27 ms and 54.6 ± 28.6msec for observer 2.

**Figure 1:**
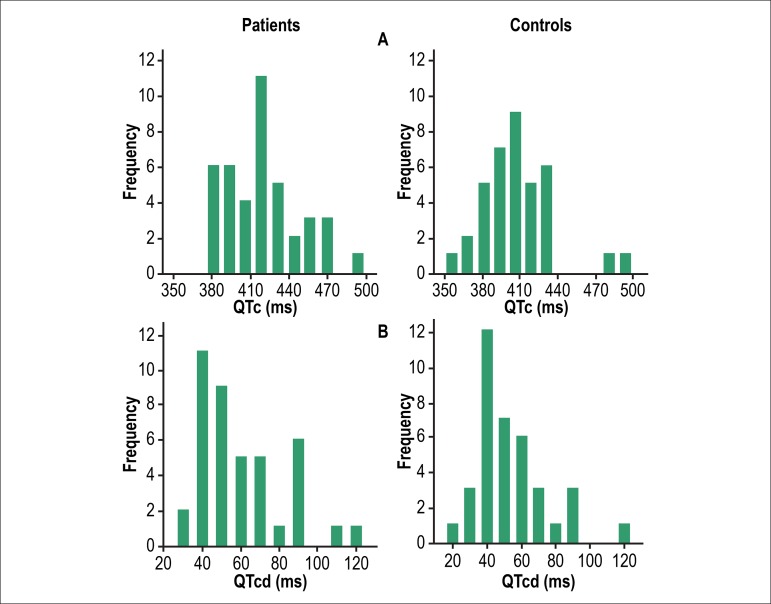
Frequency distribution of corrected QT interval, QTc (panel A) and dispersion of QTc, QTcd (panel B) in 41 hemodialysis patients and 37 controls. Data refer to the mean values of the two observers.

**Figure 2:**
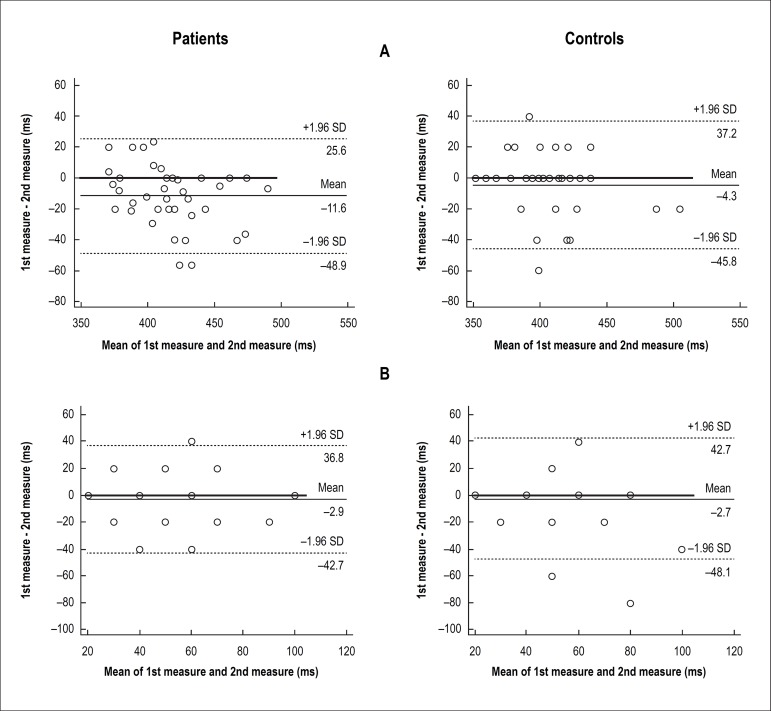
Intra-observer concordance (Bland Altman analysis of agreement) of measures of corrected QT interval, QTc (panel A) and dispersion of QTc, QTcd (panel B) in 41 hemodialysis patients and 37 controls of the study. Data refer to observer 1. Number of markers can be lower than the number of participants due to overlapping of markers.

**Figure 3:**
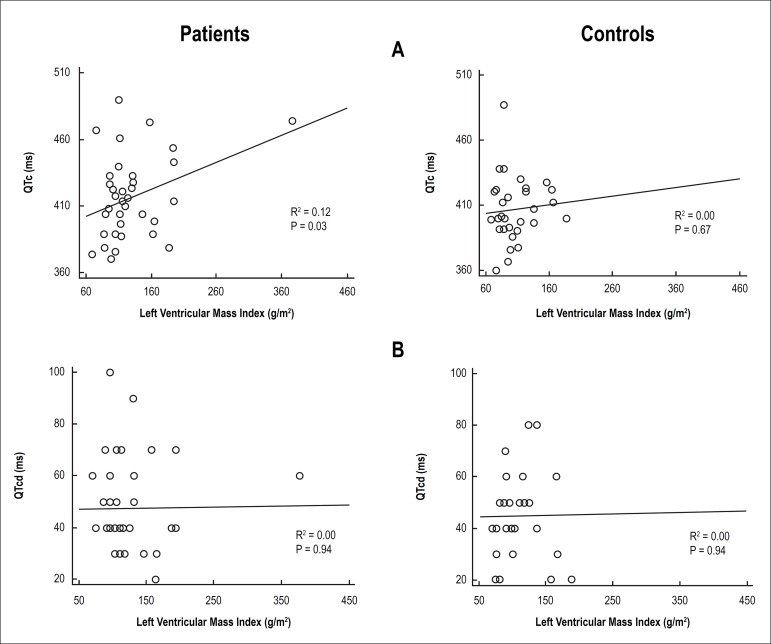
Linear regression of left ventricular mass index with corrected QT interval, QTc (panel A) and dispersion of QTc, QTcd (panel B) in 38 hemodialysis patients and thirty controls: data refer to observer 1.

Frequency distributions of both QTc and QTcd measures for patients and controls are in Figure 1. Intra and inter observer linear correlation coefficients for QTc and QTcd of HD patients and controls are in Table 2. Intra and inter observer concordance (inter-rater agreement) of measures of QTc and QTcd for each group are in Table 3. The Bland Altman diagrams addressing intra and inter observer agreement for these variables are in Figures 2 and 3, respectively.

**Table 2:** Intra- and interobserver linear correlation coefficients of QTc and QTcd in 41 hemodialysis patients and 37 controls

		Intraobserver^a^		Interobserver	
		**ρ (95% CI)**	**p**	**ρ (95% CI)**	**p**
Patients	QTc	0.83 (0.69 – 0.90)	< 0.001	0.92 (0.85 – 0.96)	< 0.001
	QTcd	0.50 (0.22 – 0.70)	< 0.001	0.72 (0.53 – 0.84)	< 0.001
Controls	QTc	0.78 (0.62 – 0.88)	< 0.001	0.82 (0.68 – 0.90)	< 0.001
	QTcd	0.39 (0.07 – 0.63)	0.017	0.50 (0.22 – 0.71)	0.001

a observer 1; QTc: corrected QT interval; QTcd: dispersion of QTc; ρ: Pearson correlation coefficient.

**Table 3:** Intra- and inter-observer concordance (inter-rater agreement) of measures of QTc and QTcd in 41 hemodialysis patients and 37 controls

		Intraobserver^a^	Interobserver
		**ĸ (95% CI)**	**ĸ (95% CI)**
Patients	QTc	0.66 (0.36 – 0.96)	0.83 (0.60 – 1.00)
	QTcd	0.14 (–0.21 – 0.49)	0.44 (0.17 – 0.70)
Controls	QTc	1.0 (1.0 – 1.0)	0.78 (0.38 – 1.00)
	QTcd	0.37 (–0.07 – 0.80)	0.32(–0.01 – 0.66)

a Observer 1; ĸ: Cohen's Kappa coefficient; QTc: corrected QT interval; QTcd: dispersion of QTc.

The association of QTc or QTcd with LVH was evaluated by linear regression analysis (Figure 3). In HD patients, a poor but significant correlation was found between values of QTc interval and LVMi (R^2^ = 0.12; p = 0.033). In contrast, no correlation was present between values of QTcd and LVMi (R^2^ = 0.00; p = 0.940). For the control group, the correspondent values were R^2^ = 0.00; p = 0.67 and R^2^= 0.00; p = 0.94, respectively.

## Discussion

LVH is a frequent abnormality and a marker of cardiovascular events and death in HD patients.^1,2^ Although alterations in QT are also associated with overall mortality and cardiovascular events in the general population,^4,15^ studies correlating LVH and changes in QT in HD patients are scarce. In the present study, we analyzed the reproducibility and reliability of QTc and QTcd measurements and their relationship with LVH as diagnosed by ECO in HD patients and in a control group. For this purpose, we resorted to a database derived from a study in which HD patients suitable to engage in an exercise treadmill test were enrolled.^8^ Mean age, gender distribution, skin color, and body mass index of patients and controls were similar. Since some diabetic patients were judged as not apt to undergo an exercise treadmill test, this may in part explain why diabetic patients have a low representation in our sample when compared to national data^16^ and to international series.^17^ In agreement with the majority of reported series, a notable number of the HD patients were in use of blood pressure drugs.^16,17^ Serum levels of C-reactive protein were greater in HD patients, which are well recognized for their chronic inflammatory state.^18^ It should be pointed out that HD patients had a standard dialysis treatment as evidenced by their mean URR and mean hemoglobin levels.^19^

The prevalence rate of LVH found in the ECO of HD patients (71%) is consistent with previous report^1^ and tended to be higher than in controls (46%), also in accordance with a previous study^20^. In contrast, left ventricular systolic function was similar in both groups, perhaps due to our recruitment criteria that privileged healthier patients able to undergo an exercise test.

Mean QTc and QTcd in our sample were lower than the ones reported in major international studies on HD patients^21-26^. Again, one of the reasons that could account for this difference was our enrollment criteria, which excluded patients with overt heart failure who are more prone to QT alterations. In support of previous reports, the mean values for QTc and QTcd as well as the frequency of enlargement in each of these two parameters tended to be higher in HD patients than in controls.^27^ Other possible explanation for the discrepancy of our results in comparison to literature may reside in the methodology chosen for the measurement of the QT and the moment the ECG was performed. We decided not to use the traditional Bazett formula to calculate heart-rate-corrected QT. The decision was taken to comply with the current recommendations of ECG interpretation^12^ which explicitly discourage the use of Bazett formula because of its inability to properly correct the QT for heart rate.^7^ It has long been known that the use of Bazett formula overestimate QT at fast heart rates and underestimate it at low heart rates^7^. A recent well designed study found that the Hodges formula is associated with lower QTc variability over the whole range of the investigated heart rates and seem to be the most accurate in determining the correct QTc.^28^ For measuring the QT, we preferred to use the tangent technique rather than the conventional methodology^11^. A study conducted in a central ECG laboratory conclude that when ECGs are interpreted by trained readers using sophisticated on-screen tools and high quality digital ECGs recorders, the results are comparable for the tangent and the conventional method. However, the QT measured by the tangent method may be shorter than the conventional method by up to 10 milliseconds.^29^ When QT measurements were manually evaluated by inexperienced readers on prints of 12-lead ECGs, the results were favorable to the method of tangent.^11^ Furthermore, we chose to record the electrocardiogram in the interdialytic period, instead of during the HD procedure, in contrast to most of the studies that addressed the relationship between electrolyte disturbances and QT changes.^23,24,26^

When looking at the pattern of frequency distribution of QTc, it can be realized that baseline values of patients are higher than controls. Accordingly, mean values of QTc tended to be higher than in the control group. These findings are consistent with other studies and may be related to the higher prevalence of LVH and electrolyte imbalance in HD patients.^30^ In contrast, distribution of QTcd looked frequencies very similar for patients and controls. It should be mentioned that many drugs, including some anti-hypertensive medications, are known to prolong the QT.^31,32^ Of note, the frequency of use of clonidine and alpha-blockers was higher in HD patients than in the controls and could potentially account for the differences in QT between groups. However, when consulting a website that is thought to be an excellent source of information regarding drugs that may affect the QT interval,^31^ such medications were not found in any of the four listed categories.^33^

The main purpose of our study was to address the reliability and reproducibility of QTc and QTcd measurements. A good correlation was found for the intraobserver measurements of QTc values in both, patients and controls. However, the intraobserver correlation of QTcd values for the two groups was poor. The interobserver values followed the same trend but, as a whole, correlation tended to be a little bit better than for the intraobserver measures probably because the mean of the two measures made by each observer was used for comparisons.

Values of kappa coefficient showed a strong intra- and interobserver agreement for QTc values and a weak one for QTcd for both patients and controls. In the Bland-Altman plots, our results showed concordance between measures of QTc, except in the intraobserver analysis of patients group. For QTcd we found a biased proportion in interobserver analysis of control group and absence of concordance in interobserver analysis of patients’ group. In summary, we found that QTcd results for reproducibility and reliability were significantly poorer than QTc, in accordance to previous reports in healthy subjects,^34,35^ patients with cardiovascular disease,^36^ or undergoing HD^37^ discouraging the use of QTcd routinely. In contrast, QTc seems to be a reliable and reproducible measure.

A linear regression was applied to assess the relationship of QTc and QTcd with LVH. In patients, a poor but significant correlation was found between values of QTc interval and LVMi and no correlation was found for QTcd. In the control group, there was no correlation between values of either QTc or QTcd interval and LVMi. The absence of correlation between LVH and QTc in the control group could be accounted for by the fact that we enrolled volunteers assigned to undergo an exercise treadmill test and had a high chance to have coronary artery disease. Predisposing risk factors for QTc prolongation include advanced age, left ventricular hypertrophy, heart failure, myocardial ischemia, hypertension, diabetes mellitus, elevated serum cholesterol, high body mass index, slow heart rate, electrolyte imbalance (including hypokalemia and hypomagnesemia) and drugs.^38^ In the control group, in which the prevalence of LVH was not as high as in HD patients, ischemic alterations may have prevailed upon muscle hypertrophy as mechanism affecting repolarization.

The link between LVH and prolonged QT found in HD patients in the present study has previously been demonstrated by a number of authors in patients with hypertension and hypertrophic cardiomyopathy^13^ and also in HD patients.^21,23,24^ However, the correlation between QTcd and LVH in HD patients is uncertain with some studies reporting positive correlation^14,21,24^ and others, corroborating our findings, absence of correlation between these variables.^22,30^ The current review of literature on the electric heterogeneity in LVH allows us to conclude that electrical disturbances do indicate ventricular structural abnormalities.^39^ The relationship between LVH and prolonged QTc has a rational biological basis although the cause of the phenomenon has not been completely defined. In the hypertrophic myocardium, multiple pathological changes occur, such as myocardial fibrosis, myocyte hypertrophy, cell death, and neurohormonal dysregulation that may have an important effect on QTc prolongation.^40^ The reasons for the contradictory results of the link between QTcd and LVH in the literature can probably be explained by the poor reproducibility and reliability of QTcd.

The present study carries some limitations such as the relatively small number of patients and the exclusion criteria. Further studies involving larger patient populations are needed to determine associations between alterations in QTc interval or its dispersion and LVH, and to determine the optimal time to measure these parameters (pre-dialysis, during dialysis, or after dialysis), as well as the standardization of cut off points for these parameters, techniques of measurements and correction for heart rate.

## Conclusion

In conclusion, we found that QTc interval, in contrast to QTcd, is a reproducible and reliable measure and had a weak but positive correlation with LVMi in HD patients. Our findings suggest that precision of measurement can be improved if the mean of two measures are obtained using the tangent technique and by the application of Hodges formulae to correct QT interval.
